# Proof of concept for a passive sampler for monitoring of gaseous elemental mercury in artisanal gold mining

**DOI:** 10.1038/s41598-017-16713-7

**Published:** 2017-11-28

**Authors:** Elias de Barros Santos, Paleah Moher, Stacy Ferlin, Anne Hélène Fostier, Italo Odone Mazali, Kevin Telmer, Alexandre Guimarães Brolo

**Affiliations:** 1 Institute of Science and Technology, Federal University of São Paulo, 12231-280 São José do Campos, SP Brazil; 2 Department of Chemistry, University of Victoria, PO Box 3055, V8W 3V6 Victoria, BC Canada; 3 Department of Geography, University of Victoria, V8W 3P2 Victoria, BC Canada; 4 Artisanal Gold Council, C100-633 Courtney Street., Victoria, B.C. V8W 1B9 Canada; 5 Institute of Chemistry, University of Campinas, P.O. Box 6154, 13083-970 Campinas, SP Brazil; 6 Functional Materials Laboratory – Institute of Chemistry, University of Campinas, PO Box 6154, Zip Code 13083-970 Campinas, SP Brazil

## Abstract

Mercury emissions from artisanal gold mining operations occurring in roughly 80 developing countries are a major workplace health hazard for millions of people as well as the largest contributor to global mercury pollution. There are no portable, cheap, and rapid methods able to inform workers or health practitioners of mercury exposure *on site* in remote locations. In this work, a proof of concept for a miniaturized mercury sampler, prepared by the direct reduction of gold into the porous nanostructures of Vycor glass (PVG), is introduced. Mercury retention on the PVG/Au sampler induces significant color changes, due to the formation of Au-Hg amalgam that affects the surface plasmon resonance characteristics of the material. The color change can potentially be quantified by the analysis of pictures obtained with a cell phone camera rapidly and onsite. Laboratory experiments showed the viability of using PVG/Au as passive sampler for monitoring of Hg°. PVG/Au samplers were then deployed in an artisanal and small-scale gold mining (ASGM) operations in Burkina Faso and it was able to indicate personal mercury exposures. The amount of mercury quantified in the samplers for all miners was higher than the current personal exposure limit set by the US Occupational Safety & Health Administration (OSHA).

## Introduction

Mercury is naturally present in the Earth’s biogeochemical system and it can be released to the atmosphere as gaseous elemental mercury (GEM, Hg°) from natural sources, such as volcanic and geological activities^1,2^, and from anthropogenic sources, such as artisanal and small-scale gold mining (ASGM), coal burning, cement production, and primary production of non-ferrous metals and chlor-alkali^3–5^. Emissions of Hg° from ASGM activities accounts for 37% of total global GEM emissions and it is considered the main human activity responsible for mercury pollution^6^. Two other forms of mercury are also present in the atmosphere although less abundant than Hg°: divalent mercury (Hg^2+^ operationally defined as reactive gaseous mercury (RGM)) and mercury associated with particulate matter (HgP)^7^. The current personal exposure limit (PEL) for Hg° set by the US Occupational Safety & Health Administration (OSHA) is 0.1 mg m^−3^ (~12 ppbv in air)^8^. In ASGM, gold is commonly extracted by amalgamation with mercury, and miners can be acutely exposed to mercury when done without adequate protection. This has been documented to be occurring in 80 + Countries^9^. The highest exposure occurs when the Au-Hg amalgam is heated to evaporate the mercury and obtain sponge gold doré (typically 16 to 22 karats in purity in Burkina Faso). In that procedure, the amalgam is generally heated to above 400 °C and the Hg° is either (typically) directly discharged into the atmosphere or (rarely) collected in a water trap or other condensation instrument, such as a retort^10^. According to Spiegel *et al*.^11^, about 10–15 million people work as gold miners, and approximately 30–50 million live in ASGM areas. Mercury use in ASGM is estimated to be around 1,600 tons^12^ and a combination of rising gold prices and more accurate information are likely to increase use and the estimate^9^. Studies conducted in different countries (Africa, South America, and Asia) have shown a high levels of exposure to inorganic mercury in people who work or live in gold mining areas, and high concentrations of Hg have been detected in their urine and hair^13–16^. This constitutes one of the largest environmental work hazards in the world today. Other human activities, where mercury manipulation and/or Hg-contaminated materials are processed, such as dentist offices, fluorescence lamp factories and others, also have the potential for mercury exposure of workers and the associated health consequences^8^. A sensitive mercury sampler that can be used as a cheap, rapid and *on-site* personal monitor for Hg° exposure should be a very useful tool for environmental monitoring and occupational health.

Currently, mercury personal exposure is measured using both active and passive sampler systems^17–19^. Active samplers use a pump that continuously samples a fixed flow of air. The airflow passes through a trap that collects mercury. The trap typically consists of a mercury absorbent material (e.g. gold or active carbon compounds). However, this type of sampler is expensive and impractical. They are relatively heavy, they require high power consumption for the operational of the pump, and the analysis of the collected mercury is realized in specialized laboratories. Unlike active samplers, passive samplers do not require a pump to collect polluted air. Although they often require longer sampling times compared to active samplers, they are generally less expensive, lighter, and do not require a power supply. However, also in the case of passive samplers available today, specialised laboratories and high level expertise are necessary to analyse and interpret the mercury collected by the trap^19^.

A large variety of devices have been developed for mercury monitoring in air^20–24^. Many of these materials are made of gold or silver, and the direct amalgamation process between the metal and mercury is the basis for the function of these devices. However, problems with portability, durability and limited sensitivity still persist^25^. Recent progress in nanofabrication techniques has allowed the production of a variety of highly sensitive nanomaterials that are smaller and lighter than the commercial systems^26–29^. James *et al*. reported a sensitive and reusable Hg° sensor based on gold nanoparticles with limit of detection of approximately 90 ppb in air^28^. McNicholas *et al*. reported a very sensitive Hg° sensor based in gold nanoparticles deposited on carbon nanotubes, and a detection limit of 2 ppb in air was achieved^29^. However, this new generation of mercury sensors still require regeneration steps, which often contributes to the increase of complexity and costs. As well, the fabrication techniques produced a limited quantity of samplers. Consequently, such materials become impractical for large scale field applications.

In this context, the purpose of this work was to demonstrate a proof of concept for a low-cost and sensitive Hg° vapor sampler. This sampler was portable and tailored towards field applications. Potentially, our sampler allow mercury exposure to be detected rapidly *on site*, using a regular cell phone camera equipped with a RGB analyzer application. The sampler consisted of nanogold prepared whitin porous Vycor glass (PVG) from the direct reduction of gold compound to Au^0^ inside the glass pores. PVG is a known porous, low cost material, and even one commercial PVG rod (~6.0 cm long × 0.6 cm in diameter from Corning) can be cut in forty 0.1 cm × 0.6 cm PVG discs. This brings the cost of one PVG disc down to less than U$ 2.00 (without considering mass fabrication). Each disc can be used to host nanomaterials into its porous structure, as reported^30–32^. In addition to its porous structure, with pores size distribution between 2–40 nm, PVG has a high surface area (~250 m^2^ g^−1^), consequently, it exhibits a high storage capacity^33^. The PVG/Au material synthesized here was employed as a passive sampler for monitoring of GEM exposure of gold miners in an ASGM site in Burkina Faso.

## Results

### Preparation and characterization of the PVG/Au sampler

**Figure 1:**
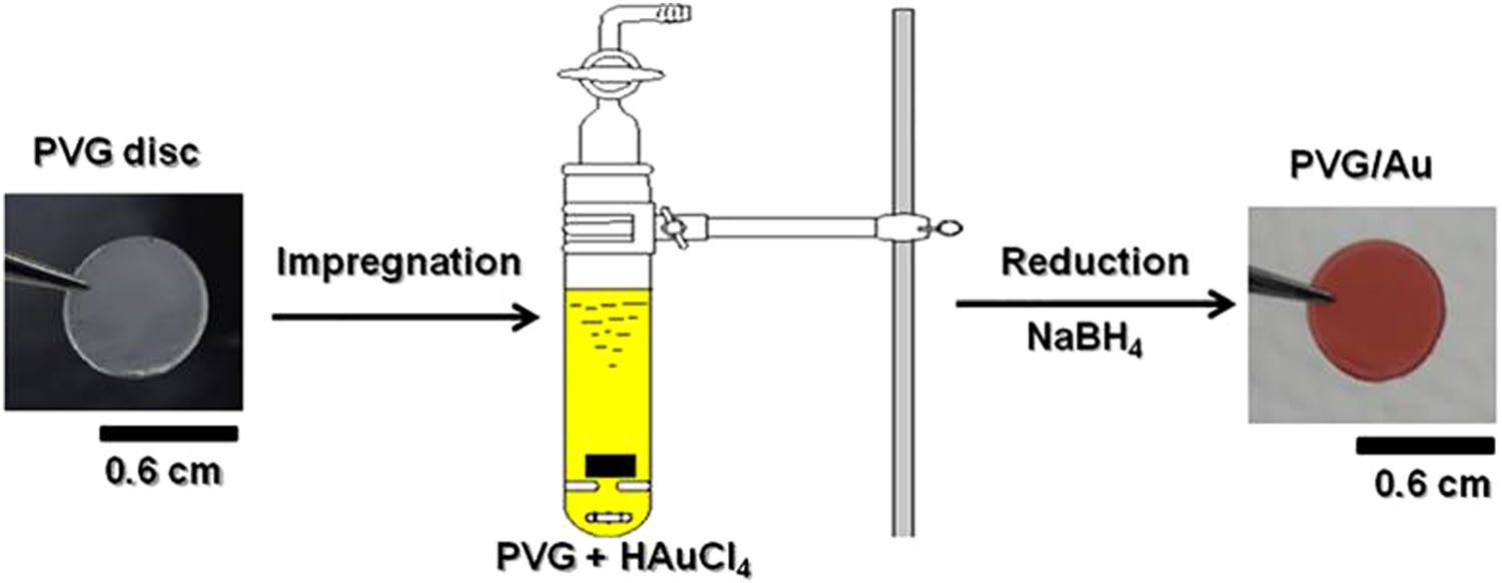
Schematic illustration of the method to prepare PVG/Au by using sodium borohydride as a reduction agent.

As shown in Fig. 1, the PVG/Au sampler was prepared in two steps: impregnation of PVG discs with HAuCl_4_ solution, followed by the direct reduction *in situ* of AuCl_4_^−^ to Au^0^, using sodium borohydride as a reduction agent. PVG has a random porous structure, with pores sizes ranging between 2 to 40 nm. The growth of gold is limited by the pore structure and nanometric gold material is formed inside the PVG. The presence of nanogold was readily detected by the red color of the PVG/Au disc, as shown in Fig. 1. Although gold is impregnated in a solid matrix of the PVG, the red color, due to the collective excitation of conducting electrons in gold nanostructures called surface plasmon resonance (SPR), is similar to what is observed in colloidal suspensions of gold nanostrucutres^34,35^.

**Figure 2:**
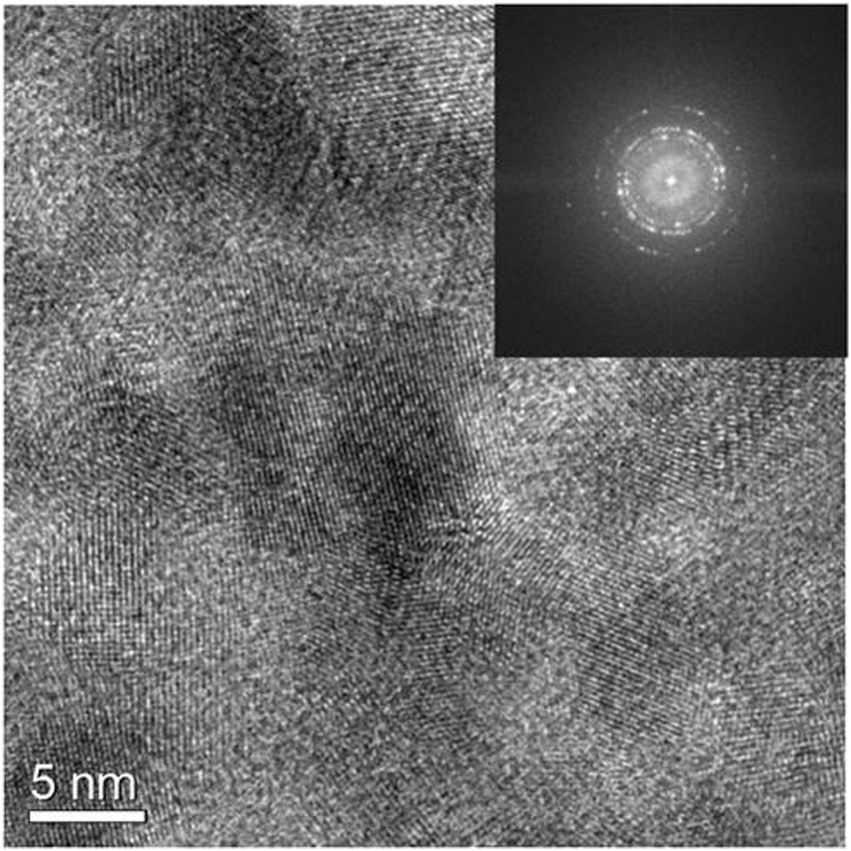
HRTEM image of crushed PVG/Au. Inset: Fast Fourier Transform (FFT) of the image.

The PVG/Au was analyzed by high-resolution transmission electron microscopy (HRTEM). The electron micrograph in Fig. 2 indicates that the PVG disc was covered by a crystalline gold thin film. This is confirmed by the fast Fourier transform (FFT) analysis of the HRTEM image (inset in Fig. 2), where the coexistence of many white spots associated to crystallinity of metallic gold nanostructures can be observed.

Many studies have shown that PVG is an excellent host matrix for nanomaterials such as semiconductor oxides and metallic nanoparticles^32,36,37^. In the present work, the amount of gold deposited in the PVG/Au discs was determined by ICP-MS. An average gold mass of 13.4 ± 4.8 μg per sample was obtained. This value corresponds to 0.04% of gold mass per PVG disc, which is much less than the storage capacity of the PVG. This result indicates that the PVG pore structure has the capacity to host more or less mass, which might be important for refining the application as a mercury sampler.

### Exposure of PVG/Au sampler to mercury vapor at room temperature

**Figure 3:**
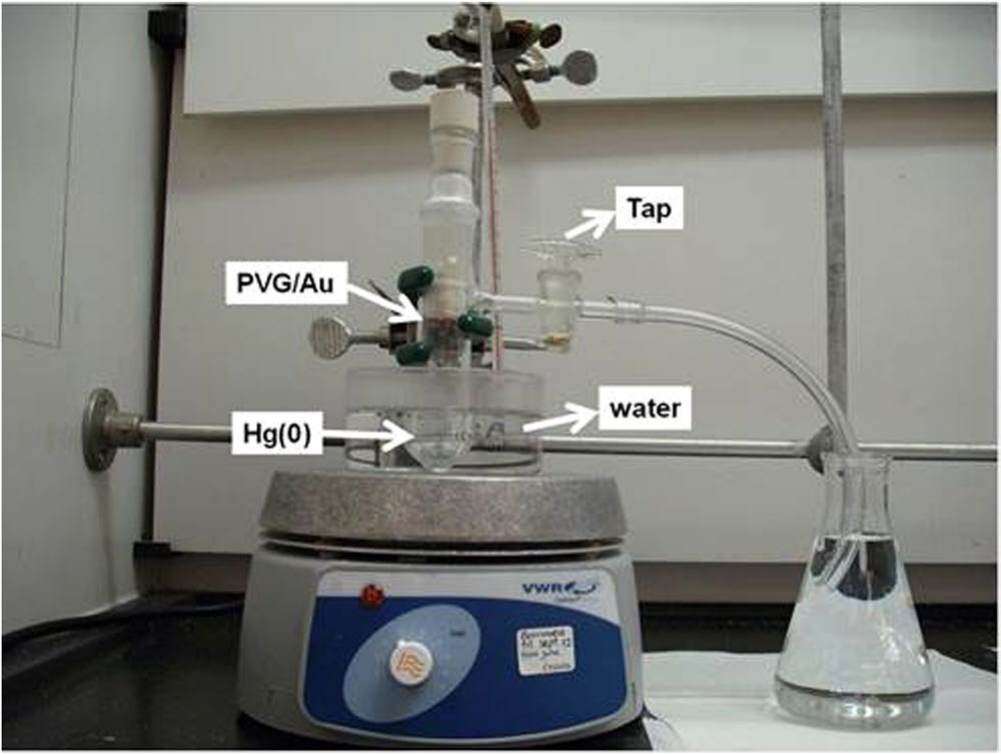
Experimental setup used to exposure PVG/Au samplers to mercury vapor in different times at 22 °C.

**Figure 4:**
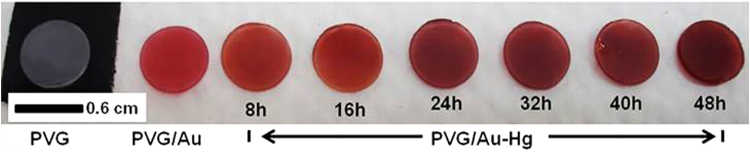
From left to right: PVG disc (colorless), PVG/Au sampler (red), and PVG/Au-Hg sampler after exposure to Hg° vapor in different times (8–48 h).

**Figure 5:**
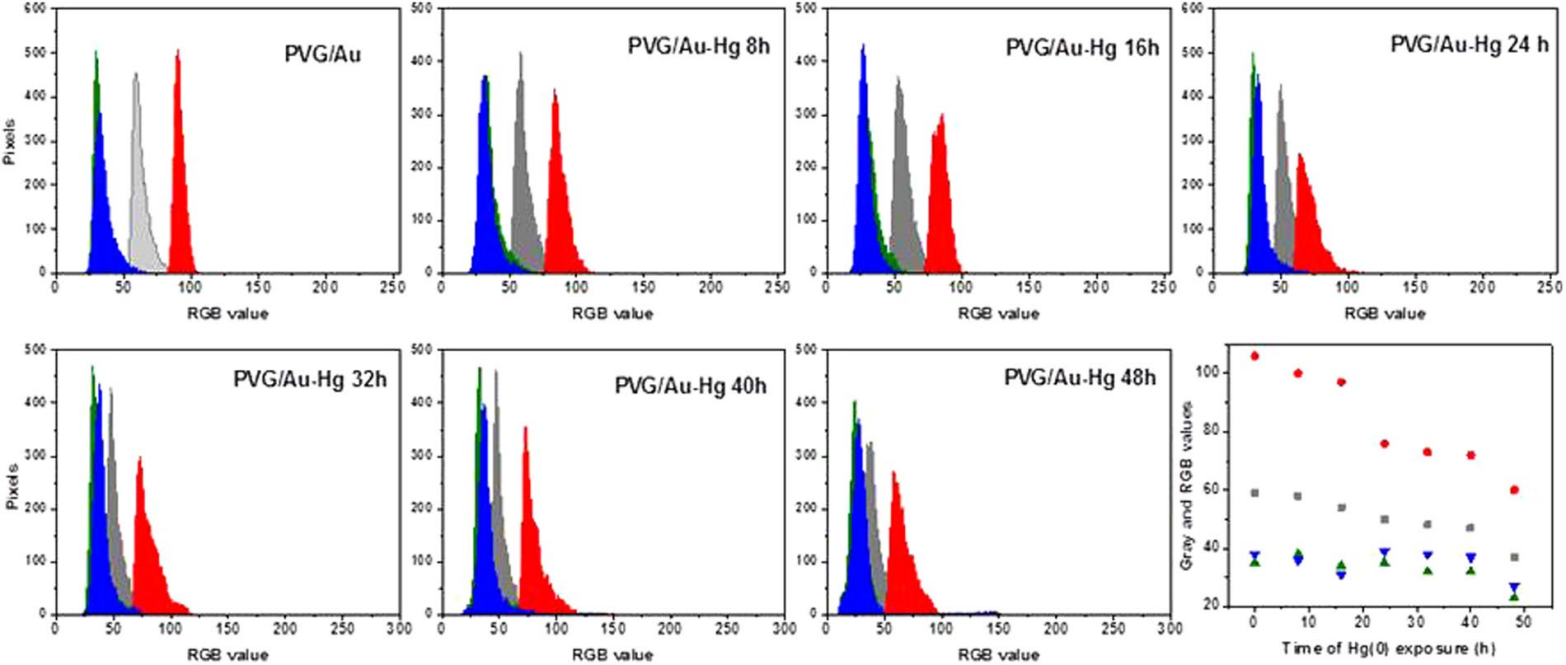
RGB and gray histograms of PVG/Au and PVG/Au-Hg after different exposure times to Hg° vapor as indicate in each spectrum. The graph on the lower-right side shows the variations trend for all RGB color channels (experiment performed with vapor pressure of Hg° at 22 °C).

Prior to field measurements, preliminary laboratory experiments were performed to provide insights into the PVG/Au sampler behavior in an atmosphere containing mercury Hg°. The PVG/Au samplers were exposed to mercury vapor using the experimental setup displayed in Fig. 3. One drop of metallic mercury was added (1.6226 g) to a 35 mL glass vessel, and six PVG/Au samplers were secured in the top part inside the vessel. The glass vessel was immersed in a water bath and the temperature was kept at 22 °C during the experiments (Fig. 3). The PVG/Au samplers were removed one-by-one at intervals of 8 h. As it can be visualized in Fig. 4, the red color tones varied for the PVG/Au samplers exposed to mercury vapour for different times. The colour change in the PVG/Au samplers after exposure to Hg° suggests the presence of mercury-gold amalgamation. The noticeable colour change reported in Fig. 4 suggests that PVG/Au samplers can be simply used as a personal monitor that indicates acute mercury exposure and with further refinement, possibly sub-acute levels. However, if quantification of the mercury exposure or if the detection of low level of exposure (which leads to subtle colour changes) is required, than this can easily be achieved using low-cost cameras currently available in portable devices, such a cell phones. For instance, cell phone photographies of the samplers from Fig. 4 were analyzed. The variaton of their RGB (Red, Green, Blue) color patterns were compared to the PVG/Au discs (used as control) that were not exposure to mercury vapor. The resulting RGB histograms are presented in Fig. 5.

Figure 5 shows that the red color channel is more sensitive to the amount of mercury then the green and blue channels. The average RGB values for the red channel shifted from 90.3 (PVG/Au, used as reference) to 58 after 48 h. It can also be observed that the red histogram becomes more asymmetric with increased Hg° exposure, leading to a decrease in pixel intensity as function of the time. There are also small variations in the green and blue histograms, and the RGB average effect is represented in Fig. 5 by the gray histogram. This RGB analysis indicates that a simple color analysis, implementable with an appropriated mobile phone application, should allow rapid evaluation of mercury sensing in field conditions for ASGM sites using the PVG/Au sampler.

**Figure 6:**
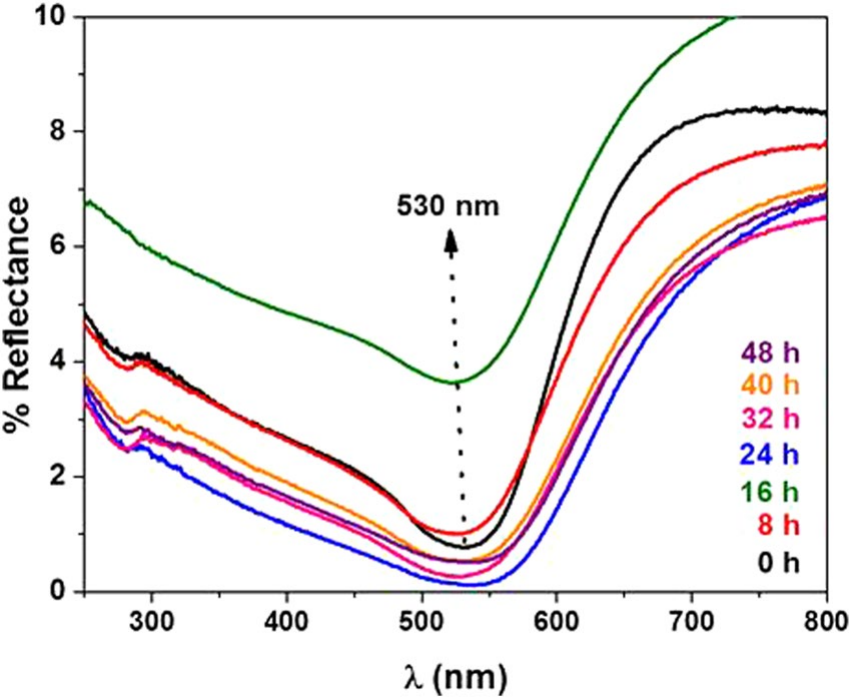
UV-vis spectra of PVG/Au sampler used as reference (black line and time 0 h) and PVG/Au-Hg samplers after different times of exposure to Hg° vapor as indicate in each spectrum with different colors (experiment performed with vapor pressure of Hg° at 22 °C).

UV-vis spectra of the samples were also obtained in reflection mode and they are presented in Fig. 6. The broad dip in Fig. 6 can be attributed to the surface plasmon resonance of the gold nanostructures embedded in PVG/Au^38^. The SPR band position at ~530 nm agrees well with the observed gold nanoparticles colloidal suspensions. The SPR band broadens with the Hg exposure, indicating light absorption in a wider range of wavelengths, and, consequently, to a darkening of the sample (as seen in the pictures in Fig. 4). Notice that Fig. 6 was obtained using a commercial UV-Vis system that only illuminates a small portion of the sample. Therefore, the %reflectance did not follow the concentration trend as the RGB (which provides a better average of the optical characteristics of the whole sample).

The vapor pressure of Hg° at 22 °C, conditions of the experiments for Figs 4–6, is 2.031 × 10^−7^ MPa. Assuming an ideal gas and using the equation presented by Huber *et al*.^39^, an ideal gas density of 8.278 × 10^−8^ mol L^−1^ is obtained. This value is equivalent to 16.6 ng mL^−1^ (2.0 ppm_v_). Although 2.0 ppm can be considered a high concentration of mercury, the main idea of this first test was to understand the behavior of PVG/Au sampler in a mercury atmosphere and also the validation of fast and simple analysis techniques such as RGB and UV-vis spectroscopy to analyze the data.

### Exposure of PVG/Au to mercury vapor at 60 °C

**Figure 7:**
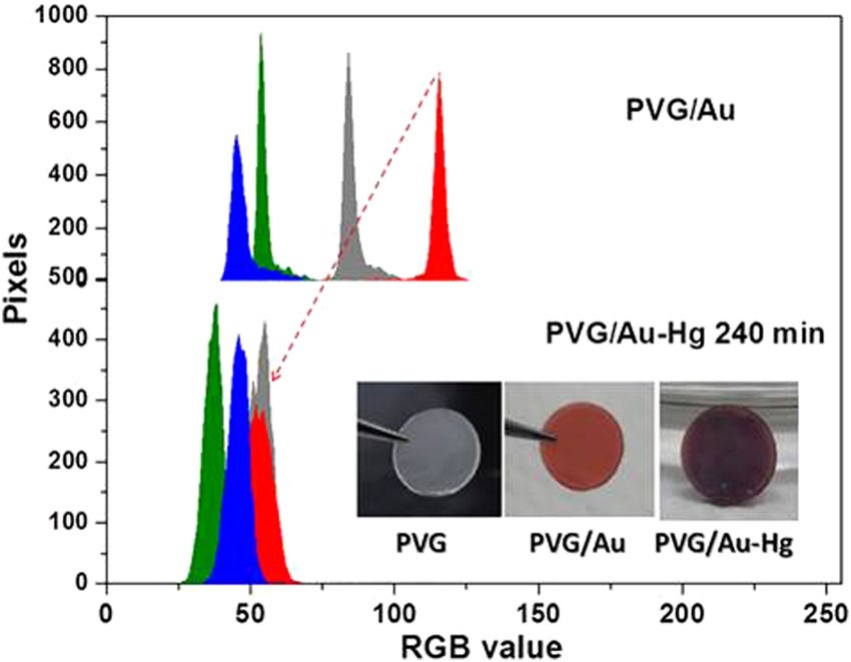
From left to right: PVG disc (colorless), PVG/Au disc (red), and PVG/Au after exposure to Hg° vapor for 240 min (PVG/Au-Hg). RGB and gray histograms of PVG/Au and PVG/Au after 240 min of exposure to Hg° (experiment performed with vapor pressure of Hg° at 60 °C).

PVG has a high surface area and it can accumulate mass inside its pore structure. Based in this information, a second experiment similar to the described in Fig. 3 was carried out, but using only one PVG/Au disc and at 60 °C. In this case, a drastic change in the color of the PVG/Au sampler was observed in Fig. 7 after 240 min of exposure to Hg° vapor at 60 °C. The colorimetric analysis of the sampler before and after exposure to Hg° indicates that the red channel is the most sensitive and its histogram shits from Red channel value from 116 to 53, respectively (Fig. 7).

**Figure 8:**
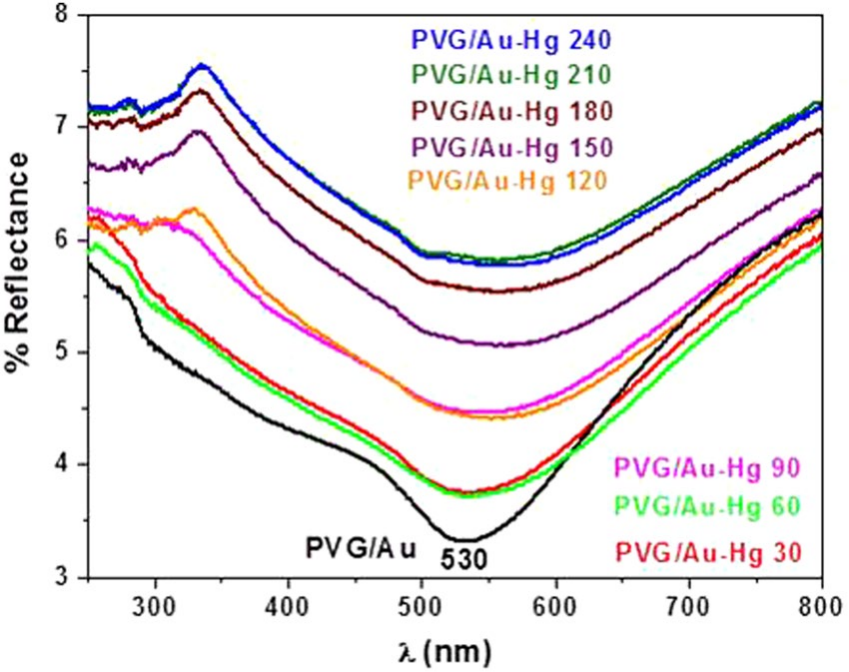
UV-vis spectra of PVG/Au before and after Hg° exposure for 240 min (experiment performed with vapor pressure of Hg° at 60 °C).

Analyzes of the UV-vis spectra of PVG/Au sampler as function of the exposure time to Hg° showed that the SPR band at 530 nm was practically completely damped after 240 min (Fig. 8(a)). The Hg° vapor density at 60 °C is 254 ng mL^−1^. The results obtained for this test indicates that the highest changes in the SPR band and in the histograms, compared to the test at 22 °C, are related to the changes in nanogold characteristics due to a higher retention of mercury. The tap in the experimental setup, indicated in Fig. 3, was kept open during the experiment to produce a flux of Hg° vapor. This result indicates that the PVG/Au sampler has a high storage capacity of mercury and it saturates only on drastic conditions (very high amounts of mercury), which is beyond of the experimental conditions tested in the present study. This also illustrates the samplers potential for detecting acute levels of mercury exposure.

**Figure 9:**
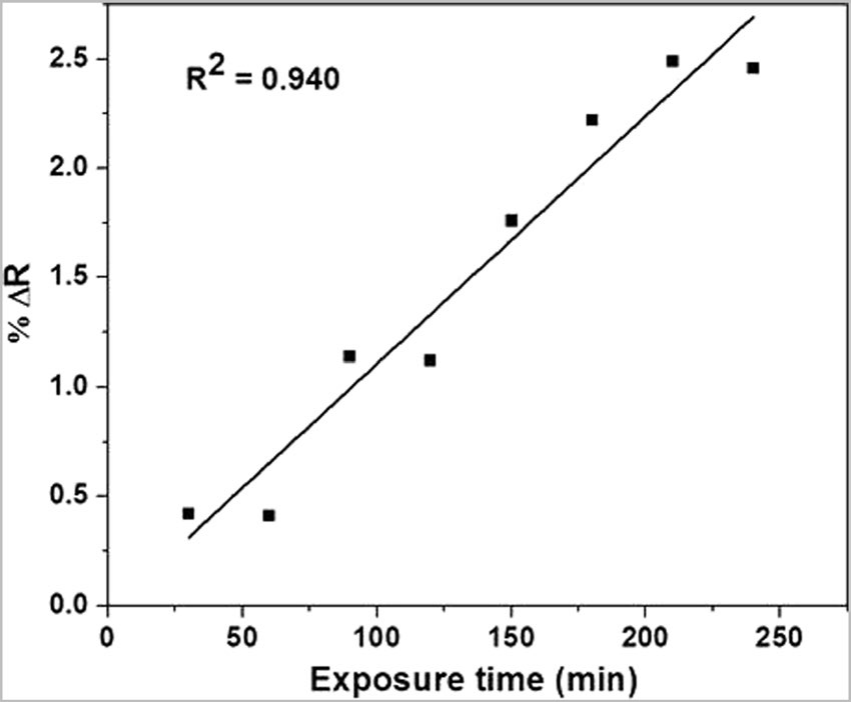
Variation of the PVG/Au reflectance before and after Hg° exposure for 240 min (experiment performed with vapor pressure of Hg° at 60 °C).

A relatively good correlation was obtained between the variation of reflectance and the exposure time (Fig. 9). This result indicates that the PVG/Au sampler is effectively sensitive to the different concentrations of mercury as a function of the exposure time, and a liner correlation can be achieved in controlled experimental conditions.

### Monitoring of mercury using PVG/Au samplers in an ASGM site in Burkina Faso

The performance of the PVG/Au sampler was also evaluated in a simple field application in an ASGM in Burkina Faso. In this case, a burning of Au-Hg amalgam was performed inside an open structure with a thatched roof. A PVG/Au sampler was placed on the front lapel of the shirt of each of the seven miners who were present at the Au-Hg amalgam burn, and one sampler (N 5) was placed on the thatched roof above the miner conducting the burn. This burning procedure is part of the normal working conditions for miners in ASGM. The miner responsible for the burn used a propane torch to heat approximately 16 g of Au-Hg amalgam in a metal bowl, releasing approximately 8.0 g of Hg° into the surrounding air within the thatched structure. The PVG/Au samplers were collected from each of the miners immediately after the burn and placed in separate glass vials with a tight fitting lid, labeled with the time, date, and the miner’s position relative to the burner. The PVG/Au samplers were numbered and their characteristics are reported in Table 1. Table 1 also shows the amount of gold in each PVG/Au sampler and the amount of retained mercury per sampler. The values in Table 1 show that the PVG/Au sampler can detect the presence of Hg in the range between ~0.06 to 0.6 μg. This is comparable to the low limits of commercial inorganic mercury samplers from SKC (quantitative determination of mercury quoted between 0.04 to 30 µg - http://www.skcltd.com). Further refinements in design can potentially increase the dynamic range of the PVG/Au samplers.

**Table 1:** Total amount of gold and mercury in the PVG/Au samplers.

PVG/Au sampler	Au amount (μg)	Hg amount (ng)	Hg:Au ratio (ng:μg)	Distance from burner (cm)	∆ Red Channel
N 1	9.79	554.7	56.66	0	81
N 2	12.99	128.5	9.89	92	56
N 3	9.33	124.5	13.34	107	18
N 4	11.34	28.9	2.55	162	36
N 5	12.07	36.5	3.02	229	31
N 6	15.92	72.6	4.56	244	19
N 7	19.53	30.3	1.55	311	5
N 8	7.80	63.4	8.13	488	109

**Figure 10:**
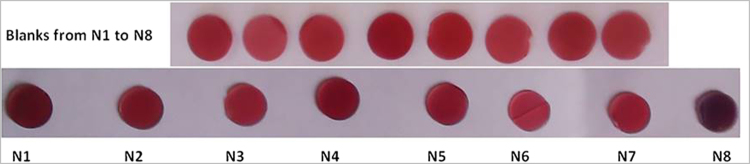
PVG/Au samplers before (blanks for comparision) and PVG/Au samplers after their application in the ASGM during the burning of Au-Hg amalgam.

**Figure 11:**
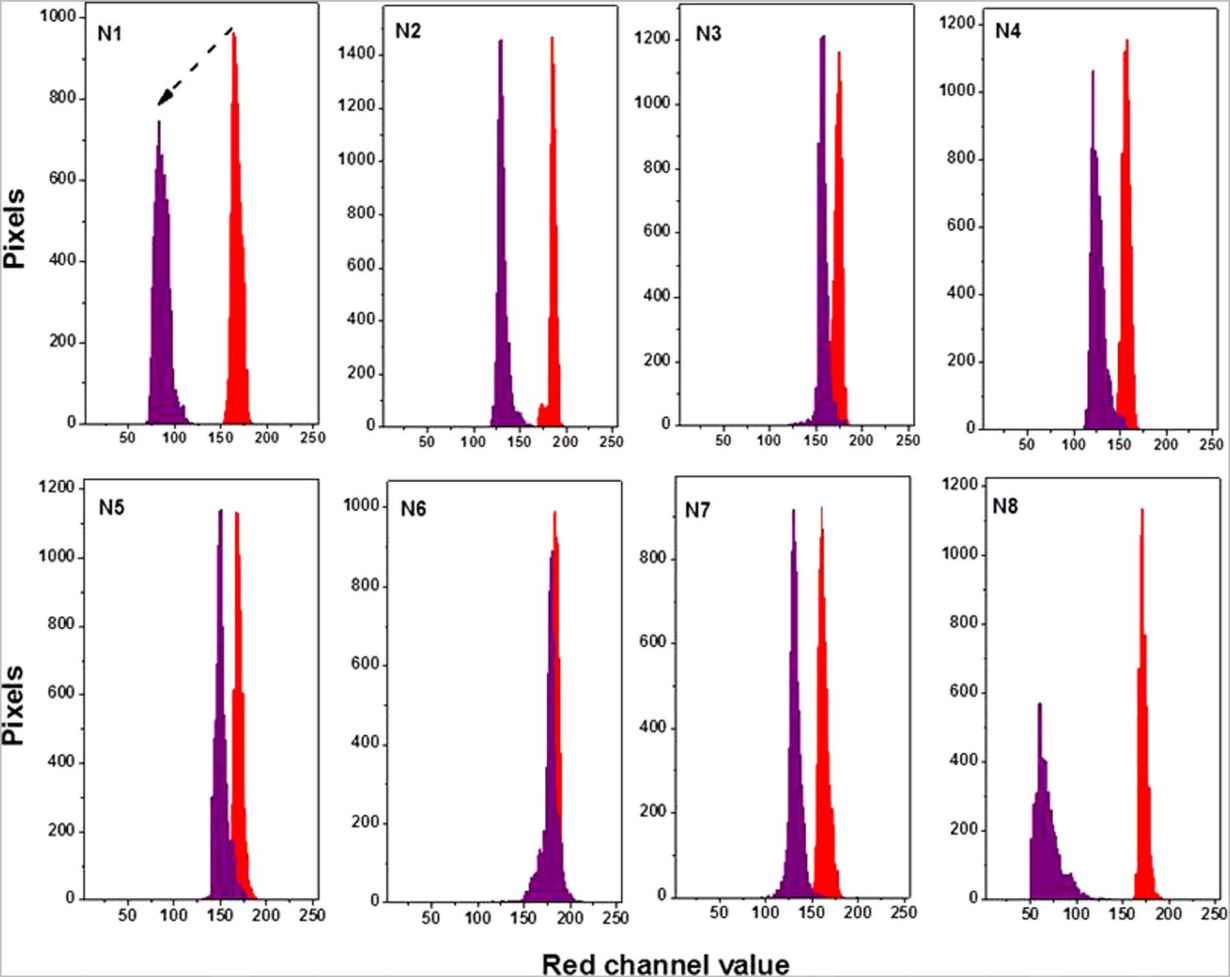
Red channel histograms of PVG/Au samplers before (red histograms) and after exposure to Hg° (purple histograms). These variation are shown in Table 1.

Figure 10 shows the PVG/Au samplers before and after the field test. Simply visual inspection of Fig. 10 shows that the red tone of the samples changed after the burning. The Red channel variation for those samples are shown in Table 1. Although the PVG/Au N 8 presented more drastic color change than the others samplers, all samples detected some level of Hg° exposure. The color change should be related to the relative amounts of mercury and gold in the sampler. In this preliminary proof of concept of the field application capabilities, neither the mass of gold (13.4 ± 4.8 μg per sample) nor its particle size distribution in the samplers was tightly controlled, which may contribute to some fluctuations in the dataset. Figure 11 shows the shift in the red channel, which were easily determined from a cell phone and demonstrate the potential of the device to detect Hg° exposure.

**Figure 12:**
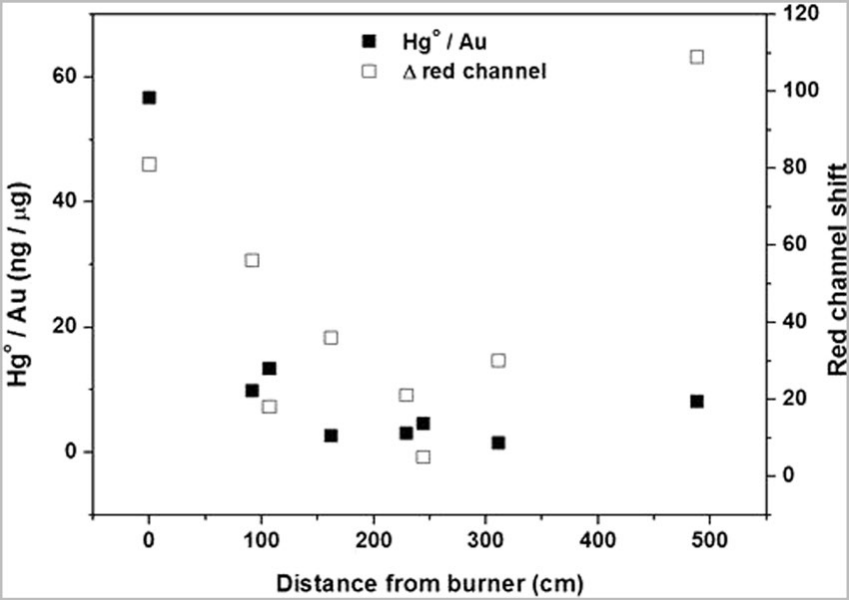
Retention of mercury per PVG/Au sampler and red channel shift as a function of the distance (cm) from the person burning the Au-Hg amalgam. The concentration in ng µg^−1^ was obtained by dividing the Hg amount detected by the gold content in each sampler.

To obtain quantitative data of the mercury retention, both Au and Hg in each sampler were quantified in the lab by using Direct Mercury Analysis (DMA) and ICP-MS techniques, respectively (Table 1). The obtained results from the field test showed that the distance of the PVG/Au sampler from the burner was the main factor controlling the relative concentration of mercury in the samplers. The highest relative amount of mercury was detected on the PVG/Au sampler held by the burner (N1), 554.7 ng of Hg, and the retention of mercury exponentially decreased with the distance (Fig. 12). The same trend was observed for the red channel shift, except for the more distante point (N8) for which the value of the shift was surprisingly high. When this point is treated as an outlier and removed, a relatively good correlation (R^2^ = 0.5962) appears between the value of the red channel shift and the Log (Hg°/Au), indicating that the sampler is effectively sensitive in terms of colorimetric sensing to low concentrations of mercury and that the color change can be used as a qualitative indicator of mercury retention. This result indicates that among the miners who are working in the gold extraction, the worker who is burning the Au-Hg amalgam is the most exposed to mercury vapor, as expected, and that mercury concentrations in the air released by amalgam burning decrease exponentially away from the source.

Several other studies have proposed alternative difusive or active measurements for GEM detection^18,24,40^. In some of them, low-levels of detection of the order of nanograms have been achieved. The direct comparision of the PVG/Au sampler elaborated in this work with other samplers reported in the literature is not as simple due to several factors, including differences in preparation methods, deployment time, experimental conditions and so on. However, as it can be observed from the laboratory and field tests presented here, the PVG/Au sampler was sensitive to mercury levels around nanograms, which is similar to the detected levels reported in the literature.

## Discussion

A proof of concept for a portable passive sampler for monitoring of GEM exposure in ASGM has been demonstrated. Results from laboratory experiments showed that the monitoring of mercury by the passive PVG/Au sampler can be realized by either UV-vis spectroscopy or by analyzing the color channels of photos of the samplers. The PVG/Au sampler was very sensitive to vapor of mercury, due to changes in the SPR characteristics of the material. Moreover, by employing the RGB color analysis of the pictures of the PVG/Au samplers, before and after exposure to mercury vapor, it was demonstrated that the red channel is the most sensitive to mercury intake by the device. In other words, when the Au-Hg amalgamation takes place, the red color of the PVG/Au samplers turns to other tones, and even achieves a purple-bluish colour depending on the amount of mercury retained by the sampler.

Results from a field experiment an an ASGM site showed that the PVG/Au sampler is also efficient as a passive sampler for outdoor monitoring of personal exposure to GEM. Potentially, the PVG/Au sampler can become a regular tool for both miners and health care workers investigating mercury exposure of miners, since it can acts as a personal dosimeter, which can be easily analyzed rapidly and onsite by using a cell phone. In the experiment realized at the ASGM site, we demonstrated that the retention of mercury by the PVG/Au sampler was dependent on the distance of each miner from the burner and that the retention of Hg on the sampler is proportional to the red channel shift. It was also observed that the miner who burned the Au-Hg amalgam was the most exposed to mercury vapor as expected. Mercury exposure information is a very important parameter for occupational health and education of the miners in order to raise awareness of the dangers of mercury and transition to safer operations. Another important conclusion from the field experiment is related to the amount of mercury quantified in each PVG/Au sampler, which was much less than the storage capacity of this sampler, indicating that the samplers may have application to capture emissions from several burns, if necessary. The issue of sampler-to-sampler variation can be addressed by optimizing the batch synthesis. This is an aspect that is now being addressed by our group.

## Methods

### Preparation of the PVG/Au sampler

A rod of Porous Vycor glass 7930 (PVG), obtained from Corning Glass, was cut using a diamond disc, washing with water as the working fluid, to obtain small PVG discs (0.1 cm thick and 0.6 cm in diameter). The PVG discs were soaked in HCl solution, 2.0 mol L^−1^, for 30 min. Subsequently they were wahsed with deionized water and immersed in acetone under ultrasonic agitation for 30 min. After that, the PVG discs were then placed into an oven at 550 °C for 12 h, being subsequently transferred to a desiccant and stored under vacuum. The cleaned PVG discs were weighed on analytical balance and subsequently immersed in 0.02 mol L^−1^ HAuCl_4_ aqueous solution for 8 h. After that, the PVG discs, impregnated with the HAuCl_4_ solution, were washed with deionized water and then were immersed in a solution of sodium borohydride (0.1 mol L^−1^) for 30 min. After this reduction step, the PVG discs were washed with deionized water and placed into an oven to dry at 100 °C overnight. The sample were removed from the oven at the same temperature, subsequently they were weighed and labelled PVG/Au.

### Characterization

High-resolution transmission electron microscopy (HRTEM) images were obtained using a JEOL JEM–3010 microscope (300 kV, 1.7 Ǻ point resolution). PVG/Au disc was crushed and the powder was suspending in isopropanol. Subsequently, an aliquot of the suspension was drop on a holey-carbon coated Cu grid for TEM analysis. Diffuse reflection spectrocopy (DRS) in the ultraviolete-visible (UV-vis) range were recorded on a PerkinElmer spectrophotometer EP Lambda1050. For UV-vis measurementes, the PVG and PVG/Au discs were stuck in a sample holder which was mounted in an integrating sphere spectral collector. For these measuremenets, BaSO_4_ powder was used as standard for instrumental background correction.

### Quantitation of mercury in the PVG/Au samplers by DMA after their application

Quantification of Hg in the PVG/Au samplers was performed by using a Direct Mercury Analyzer^®^ (DMA-80 TRICELL, Milestone, Italy). This equipment contains an automatic sampler that receives nickel boats (sample holders), a quartz furnace, a cobalt–manganese oxide catalyst, a gold-coated sand amalgamator and an atomic absorption detection cell with three different path lengths. The different steps of the analysis are controlled by software. The operation of the equipment is described in more detail in Santos *et al*. (2016)^41^. Calibration curves were constructed for the medium and the shorter path lengths by triple analyses of 10 to 600 μL aliquots of Hg standard solutions (10.0, 100.0 or 1000.0 μg L^−1^ depending on the desired final amount of Hg) prepared from a stock Hg standard solution (1.000 ± 0.003 mg mL^−1^) (Tec-Lab^®^ Hexis, Jundiaí, Brazil) diluted in a 10% (v/v) HNO_3_ solution. Dynamic linear ranges of calibration curves were 0.1 to 15 ng of Hg and 15 to 600 ng of Hg, for the medium and the shorter path lenghts, respectively. The limit of detection of the method was 0.4 and 13 ng of Hg and the limit of quantitation was 1.5 and 44 ng of Hg for the medium and the shorter path lenghts, respectively. Precision (n = 3) was < 3%.

For analysis, each PVG/Au was placed in a nickel boat of approximately 360 mm (L), 110 mm (l) and 110 mm (h) which was automatically inserted into the furnace under an oxygen flow, also serving as carrier gas. In this device, possible interfering species are removed onto the catalyst; the Hg(0) is selectively retained onto the amalgamator and then thermally desorbed and carried out to the detection cell; detection is performed at 253.7 nm.

### Quantitation of gold in the PVG/Au samplers by ICP-MS

To extract the gold from PVG/Au discs, the PVG/Au samplers were soaked in aqua regia solution (nitric acid and hydrochloric acid in the ration 1:3) for 12 h. The acid solutions, containing dissolved gold, was diluted using deionized water to ajust its concentration according to a calibration curve previously builted with a gold pattern. After that, the solutions were analyzed using a instrument X-Series II (X7) quadrupole ICP-MS from Thermo Scientific.
